# EZH2 Inhibition by DS3201 Triggers the Kaposi’s Sarcoma-Associated Herpesvirus Lytic Cycle and Potentiates the Effects Induced by SAHA in Primary Effusion Lymphoma Cells

**DOI:** 10.3390/v16091490

**Published:** 2024-09-20

**Authors:** Roberta Gonnella, Flavia Collura, Vincenzo Corrado, Michele Di Crosta, Roberta Santarelli, Mara Cirone

**Affiliations:** Department of Experimental Medicine, Sapienza University of Rome, Viale Regina Elena 324, 00161 Rome, Italy; roberta.gonnella@uniroma1.it (R.G.); collura.1953853@studenti.uniroma1.it (F.C.); corrado.2082106@studenti.uniroma1.it (V.C.); michele.dicrosta@uniroma1.it (M.D.C.); roberta.santarelli@uniroma1.it (R.S.)

**Keywords:** KSHV, p53, lytic cycle, autophagy, PEL cells

## Abstract

Primary Effusion Lymphoma (PEL) cells carry Kaposi’s sarcoma-associated herpesvirus (KSHV) in a latent state, except for a small number of cells in which the virus replicates to ensure its persistence into the infected host. However, the lytic cycle can be reactivated in vitro by exposing these lymphoma cells to various treatments, leading to cell lysis. To restrict viral antigen expression, KSHV induces repressive epigenetic changes, including DNA methylation and histone modifications. Among the latter, histone deacetylation and tri-methylation of Histone H3 lisyne-27 (H3K27me3) have been reported to play a role. Here, we found that the inhibition of H3K27 tri-methylation by valemetostat DS3201 (DS), a small molecule that inhibits Enhancer of Zeste Homolog 2 (EZH2) methyltransferase, induced the KSHV lytic cycle in PEL cells, and that this effect involved the activation of the wtp53–p21 axis and autophagic dysregulation. DS also potentiated the lytic cycle activation mediated by the Histone deacetylases (HDAC) inhibitor Suberoylanilide hydroxamic acid (SAHA) and reinforced its cytotoxic effect, suggesting that such a combination could be used to unbalance the latent/lytic cycle and further impair the survival of PEL cells.

## 1. Introduction

Kaposi’s sarcoma-associated herpesvirus (KSHV), a virus belonging to the gammaherpesvirus family, displays strong oncogenic properties. It is closely associated with human cancers such as Kaposi’s Sarcoma and Primary Effusion Lymphoma (PEL). KSHV induces a lifelong infection in a small percentage of the population and can establish a latency or lytic program, depending on the infected target cells. Even if the latent antigens play the major role in KSHV pathogenesis, the lytic proteins also contribute to it. Moreover, the release of viral particles is required to ensure viral persistence into the host, as KSHV-infected cells continuously undergo cell death. Among the lytic antigens involved in the oncogenic process are viral IL-6 (v-IL6), which promotes cell proliferation, angiogenesis and inflammation through the activation of Signal Transducer and activator of transcription 3 (STAT3) [1], and viral G protein-coupled receptor (vGPCR), which activates serine/threonine protein kinases AKT, sustaining the survival of endothelial cells, one of the major targets of KSHV infection together with B lymphocytes [2]. However, as KSHV replication is lytic, the virus activates it only in a small percentage of cells in vivo, keeping under tight control the latency/lytic replication switch and allowing both viral survival and carcinogenesis [3]. Viral replication initiates with the expression of replication transactivation activator (RTA) that triggers the expression of early lytic antigens such as basic region leucine-zipper protein (KbZIP) and late antigens such as p64 in a pre-established temporal order [4]. As noted above, the majority of cancer cells carry KSHV in a latent state, and the induction of a massive lytic cycle represents a strategy to reduce tumor cell survival. This can be achieved through different treatments such as 12-O-tetradecanoylphorbol-13-acetate (TPA) or by exposing cells to epigenetic drugs such as histone deacetylase inhibitors (HDACi), e.g., trichostatin A (TSA) or butyrate [5]. It is, indeed, important to highlight that epigenetic changes represent a common mechanism through which oncoviruses restrict antigen expression to maintain a latency program [6]. For example, it is known that the promoter of the RTA gene is associated with HDACs during latency, resulting in its repression. Besides deacetylation, a key role in restricting viral antigen expression is also played by DNA methylation of viral gene promoters [7]. Accordingly, DNA de-methylating agents such as 5-azacytidine (5-AZA) can efficiently trigger viral replication. Among other epigenetic changes induced by KSHV are histone methylation, even if this mechanism remains less investigated compared to the histone acetylation. Histone methylation is mediated by enzymes called methyltransferases that add one, two or three methyl groups on particular lysine substrates on histones but also on non-histone proteins (writers). This epigenetic modification is also regulated by demethylases that, instead, remove methyl groups from methylated proteins (erasers) [8]. Depending on the residues that undergo methylation and on whether histones become mono-, di-, or trimethylated (me1, me2, or me3), the outcome on gene expression may be oppositely influenced [9]. For example, trimethylation of Histone H3 lysine-27 (H3K27), Histone H3 lysine-9 (H3K9) and Histone H4 lysine-20 (H4K20) is known to be associated with the repression of genes. Among them, H3K27 methylation occurs in dynamic heterochromatin and thus is the most reversible epigenetic change [10]. H3K27 tri-methylation is mediated by Enhancer of Zeste Homolog 2 (EZH2) histone methyltransferase in a polycomb repressive complex 2 (PRC2)-dependent manner, while its demethylation is mediated by demethylases such as histone demethylase jumonji domain containing-3 (JMJD3). Interestingly, regarding KSHV, in addition to HDACs, RTA promoter is also associated with H3K27me3, a repressive mark, and with H3K4me3, that instead promotes gene expression, suggesting a complex regulation of RTA expression [11]. In addition to RTA, H3K27me3 marks are enriched in the genes encoding for late viral genes, silencing their expression during latency or early lytic phase activation. Accordingly, during lytic reactivation, H3K27 tri-methylation of the lytic gene is reduced. Therefore, increasing the demethylating activity of JMJD3 or inhibiting the tri-methylation induced by EZH2 represent strategies to induce KSHV replication [11]. Previous studies have shown that EZH2 depletion increases wild-type p53 (wtp53) stability through the de-repression of cyclin-dependent kinase inhibitor 2 A (CDKN2A) [12] and that the activation of wtp53 may, in turn, suppress EZH2 expression by repressing the EZH2 gene promoter [13]. Acetylation can also trigger the activation of p53 [14], and p53 has been shown to be involved in reactivation of KSHV from latency [15,16,17,18]. Among other numerous effects, EZH2 can also regulate autophagy via transcriptional regulation of the mechanistic target of rapamycin (mTOR) pathway [19], and autophagy activation has been found to promote Epstein-Barr virus (EBV) [20] and KSHV replication [21], even if the autophagic flux is blocked at the final phases, which is needed to prevent viral degradation into the lysosomes. Based on this background, in this study, we investigated whether valemetostat DS3201 (DS), a small molecule able to inhibit EZH2, could activate the KSHV lytic cycle in two PEL cell lines, BC3 and BCBL-1 and if this effect could involve the activation of wtp53 and autophagy dysregulation. We also investigated if lytic cycle activation in these cells could be potentiated by the combined treatment by DS and SAHA, the first HDACi introduced in clinical practice and reported to be able to activate viral replication [22].

## 2. Materials and Methods

### 2.1. Cell Cultures and Treatments

BC3 (ATCC, CRL-2277) and BCBL1 (kindly supplied by prof. P. Monini, Istituto Superiore di Sanità, Rome, Italy) are KSHV-positive PEL cell lines. They were cultured in RPMI 1640 medium (Sigma-Aldrich, Burlington, MA, USA) supplemented with 10% fetal bovine (FBS) (Sigma-Aldrich, Burlington, MA, USA), L-glutamine (2 mM) (Aurogene, Roma, Italy), and streptomycin/penicillin (100 mg/mL) (Aurogene, Roma, Italy) in an incubator at 37 °C in a 5% CO_2_ humified setting.

Cells were seeded in 6 well plates at a concentration of 3 × 105/mL and were treated for 24 h with the following chemicals: DS3201(DS) (selective EZH1/2 dual inhibitor, Selleckem; Houston, TX, USA, cat. N. S8926), suberoylanilide hydroxamic acid (SAHA) (HDAC inhibitor, Selleckem; Houston, USA, cat. n. S1047), pifithrin-α (ip53) (p53 inhibitor, Sigma-Aldrich; Burlington, USA, cat. n. 506132), bafilomycin A1 (BAF) (Selleckem, Houston, TX, USA; cat. n. S1413), 3-Methyladenine (3-MA, Selleckem, Houston, USA; cat. n. S2767), and phosphonoacetic acid (PAA) (Selleckem; Houston, TX, USA, cat. n. S3356). Chemicals were added to cell cultures at the final concentration of 1 µM (DS), 5 µM (SAHA), 20 µM (ip53), 5 mM (3-MA), and 500 µM (PAA).

Bafilomycin A1 (BAF) was added for the last 3 h at a final concentration of 20 nM. Cells were pretreated with 3-MA (5 mM), Pifithrin-α (ip53) (20 µM) or PAA (500 µM) for 1h before adding DS (1 µM) and SAHA (5 µM) and collected 24 h later. Untreated cells were used as a control group (CT).

### 2.2. Trypan Blue Exclusion Assay

A trypan blue dye exclusion assay was performed 24 h after treatments. The cell suspension was mixed with trypan blue (Sigma-Aldrich, Burlington, MA, USA; cat. n. T8154), a dye that can stain dead cells exclusively, live cells were consequentially identified and counted by light microscopy using a Neubauer hemocytometer. The experiments were performed in triplicate and repeated at least three times.

### 2.3. Immunofluorescence

The control cells and cells treated with DS, SAHA and with both chemicals were harvested after 24 h of culture, centrifuged at 1200× *g*, washed 1× with PBS, seeded onto multispot microscope slides (Thermo Scientific, Braunschweig, Germany, 2320) and finally air-dried. The cells were fixed with 2% paraformaldehyde (Electron Microscopy Science, 157-8) for 20 min, washed 3 times in PBS, permeabilized with 0.2% Triton X-100 (Sigma-Aldrich, T-8787)–PBS for 5 min at room temperature (RT), and then blocked with 3% bovine serum albumin (BSA)–1% glycine–PBS for 30 min at RT. Samples were incubated with the primary antibody (mouse monoclonal anti KBZIP dil. 1:300, Santa Cruz Biotechnology Inc., Dallas, TX, USA, cat. n. sc-69797, and mouse monoclonal anti gp64 dil 1:50, Santa Cruz Biotechnology Inc., Dallas, TX, USA; cat. n. sc-65444) for 1 h at RT. After washing in PBS, the samples were incubated with the secondary antibody (Cy3-conjugated sheep anti-mouse IgG, dil. 1:1000, Jackson Immuno Research; cat. n.515-165-062) in the dark at RT for 30 min. Finally, after three washes with PBS, the samples were stained with DAPI (Sigma-Aldrich D9564). The microscope slides were mounted using PBS-glycerol 1:1 and visualized by an Apotome axio observer Z1 inverted microscope (Zeiss Oberkochen, Germany), equipped with an Axiocam MRM Rev.3 camera at 40× magnification.

### 2.4. Western Blot Analysis 

To examine protein expression after different treatments, cells were harvested, washed with phosphate-buffered saline (PBS; Gibco, 18912-014), pelleted and finally lysed in RIPA buffer (150 mM NaCl, 1% NP-40, 50 mM Tris-HCl (pH 8), 0.5% deoxycholic acid, 0.1% SDS, protease and phosphatase inhibitors). The protein amount was determined using Quick Start Bovine Serum Albumin (BSA) assay (Bio-Rad, Hercules, CA, USA; cat. n. 5000206), and 10 μg of proteins of each sample were resolved on 4–12% NuPage Bis-Tris gels (Life Technologies, Carlsbad, CA, USA) according to the manufacturer’s instructions. After electrophoresis, proteins were then transferred to nitrocellulose membranes (Amersham, Buckinghamshire, UK; cat. n. 10600002) for 45 min in tris-glycine buffer. After 30 min blocking in 1× PBS-0.1% Tween20-3% BSA (Applichem, GMBH, Darmstadt, Germany; cat. n. A1391,0100), the blots were probed with specific primary antibodies overnight at 4 °C. The membranes were subsequently washed three times with 1× PBS-0.1% Tween20 and probed with the suitable secondary antibody HRP-conjugated at a dilution of 1:10,000 for 30 min at RT. After three further washes, the bands were visualized by ECL (Advansta, San Jose, CA, USA; cat. n. 12045-D20).

### 2.5. Antibodies

The primary mouse monoclonal antibodies used in Western blots were as follows: mouse monoclonal anti-KBZIP (1:500) (Santa Cruz Biotechnology Inc., Dallas, TX, USA; cat. n. sc-69797), mouse monoclonal anti-p53 (1:300) (Santa Cruz Biotechnology Inc., Dallas, TX, USA; cat. n. sc-126), and mouse monoclonal anti-H3K27me3 (1:1000) (Sigma-Aldrich, St. Louis, MO, USA; cat. n. 05-1951-S). The rabbit polyclonal antibodies used in Western blots were the following: anti-PARP (1:1000) (Cell Signaling, Danvers, MA, USA; cat. n. 9542), rabbit polyclonal anti-p21 (1:500) (Cell Signaling Danvers, MA, USA; cat. n. 2947), and rabbit polyclonal anti-LC3I/II (1:1000) (Novus, Littleton, CO, USA; cat. n. NB100-2220). To assess loading controls, the blots were probed with the following antibodies: mouse monoclonal anti-GAPDH (1:10,000) (Santa Cruz Biotechnology Inc., Dallas, TX, USA; cat. n. 47724), rabbit polyclonal anti-Histone H3 (1:500) (Cell Signaling Danvers, MA, USA; cat. n. 9715), and mouse monoclonal anti-β-Actin (1:10,000) (Sigma Aldrich, St. Louis, MO, USA; cat. n. A5441). Goat anti-mouse IgG (Sigma-Aldrich, 401215, Burlington, MA, USA), and goat anti-rabbit IgG (DC03L; Sigma-Aldrich, Burlington, MA, USA), both conjugated with horseradish peroxidase, were used as secondary antibodies. All the primary and secondary antibodies were diluted in 1x PBS-0.1% Tween20 solution containing 2% of BSA.

### 2.6. Real-Time Quantitative PCR (RT-qPCR)

Total RNA from the PEL cell lines treated with DS (1 mM) for 24 h and from untreated cells (CT) was extracted by using TRIzolTM Reagent (Thermo Fisher, Waltham, MA, USA; cat. n.15596026) according to the manufacturer’s instructions and was subjected to DNAse treatment (Norgen BioteK Corp.) for 10 min at RT to remove DNA contamination. After in vitro reverse transcription, quantitative Real-Time PCR (RT-qPCR) was carried out by using SensiFast SYBR No-ROX kit (Bioline; cat. n. BIO-94005). All values were normalized to β-actin (endogenous gene controls). 

The primers used were as follows:

RTA Fw-5′-TGTAATGTCAGCGTCCACTC-3′

RTA Rv-5′-ATTGCTGGGCCACTATAACC-3′

K8.1 Fw-5′-AAAGCGTCCAGGCCACCACAGA-3′

K8.1 Rv-5′-GGCAGAAAATGGCACACGGTTAC-3′

Actin Fw-5′-TCATGAAGTGTGACGTGGACATC-3′

Actin Rv-5′-CAGGAGGAGCAATGATCTTGATCT-3′

### 2.7. Densitometric Analysis 

Bands derived by Western blots were quantified by densitometric analysis through ImageJ software, which was downloaded from the NIH website (available online: http://imagej.nih.gov, accessed on 6 June 2024).

### 2.8. Statistical Analysis

Results are represented by the mean plus standard deviation (SD) of at least three independent experiments, and statistical analyses were performed with Graphpad Prism^®^ software (version 9; Graphpad Software Inc., La Jolla, CA, USA). Student’s *t*-test was used to demonstrate statistical significance. The difference was considered statistically significant when the *p*-value was: * < 0.05; ** < 0.01. 

## 3. Results

### 3.1. Enhancer of Zeste Homolog 2 (EZH2) Inhibition by Valemetostat DS3201 (DS) Triggers Kaposi Sarcoma Associated Herpesvirus (KSHV) Lytic Cycle Activation in Primary Effusion Lymphoma (PEL) Cells

**Figure 1:**
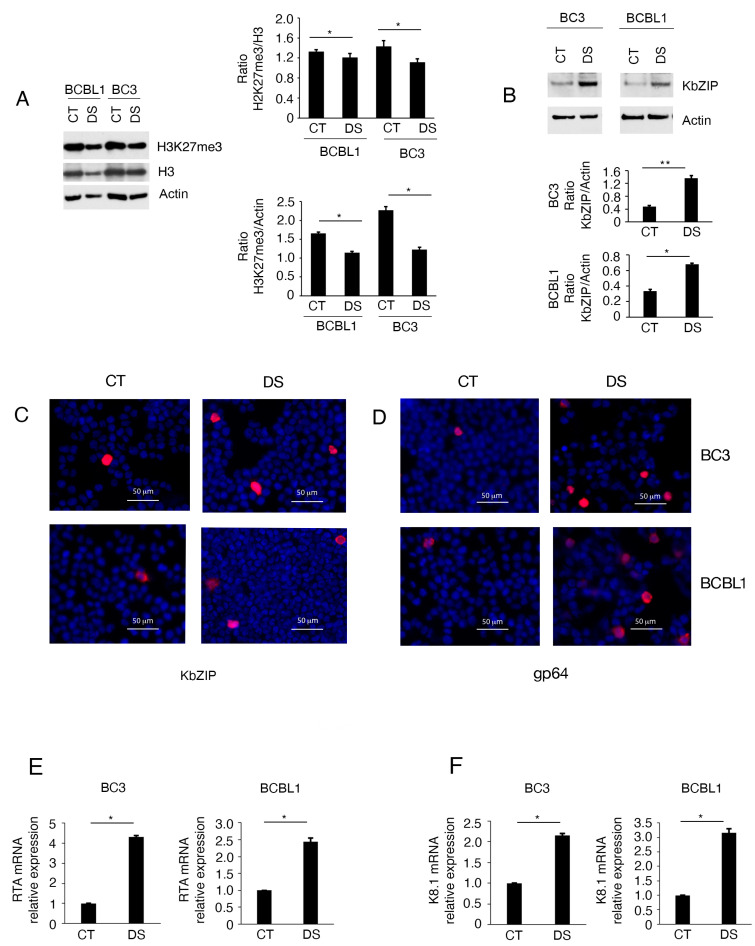
Valemetostat DS3201 (DS) activates Kaposi sarcoma associated herpesvirus (KSHV) lytic cycle in BC3 and BCBL-1 PEL cells. BC3 and BCBL-1 were exposed to DS3201 (DS) for 24 h, and (**A**) H3K27 trimethylation and (**B**) basic region leucine-zipper protein (KbZIP) expression were evaluated by Western blot analysis. Bar-graphs represent the mean plus SD of proteins of interest on house-keeping proteins calculated based on three different experiments; (**C**) KbZIP and (**D**) gp64 expression, as evaluated by IFA in BC3 and BCBL-1 cells treated by DS, Bar = 50 μM. The percentage of positive cells was 10% for KbZIP in both BC3 and BCBL-1, and 12% and 15% for gp64 in BC3 and BCBL-1, respectively); (**E**,**F**) Replication transactivation activator (RTA) and K8.1 mRNA expression as evaluated by Real Time quantitative PCR (RT-qPCR). The difference was considered statistically significant when the *p*-value was: * < 0.05; ** < 0.01.

We evaluated if valemetostat DS3201 (DS), a small molecule able to inhibit Enhancer of Zeste Homolog 2 (EZH2) activity, could trigger the lytic cycle activation of KSHV in BC3 and BCBL-1, PEL cells that harbor the virus in a latent state. To assess DS activity, we investigated the reduction of H3K27me3 in PEL cells (Figure 1A). Similar to what has been reported for JSC1 [11], a PEL cell line double-infected by Kaposi sarcoma associated herpesvirus (KSHV) and Epstein-Barr virus (EBV), we found that the KSHV lytic cycle was induced in both BC3 and BCBL-1 cells after 24 h of treatment at a 1 µM dose, based on the expression of early lytic antigen basic region leucine-zipper protein (KbZIP) as assessed by Western blot analysis (Figure 1B). These results were confirmed by IFA experiments, in which we observed that cells expressed KbZIP (encoded by K8) (Figure 1C), and by Real Time quantitative PCR (RT-qPCR), in which we found that DS-treated cells express RTA and K8.1 (Figure 1E,F). Furthermore, the expression of the late lytic antigen p64 (encoded by K8.1) was induced by DS in PEL cells (Figure 1D), suggesting that EZH2 inhibition led to a complete lytic cycle activation in these cells. 

### 3.2. EZH2 Inhibition by DS Activates wtp53, Which in Turn, Inhibits EZH2 Activity, Sustaining KSHV Lytic Antigen Expression

**Figure 2:**
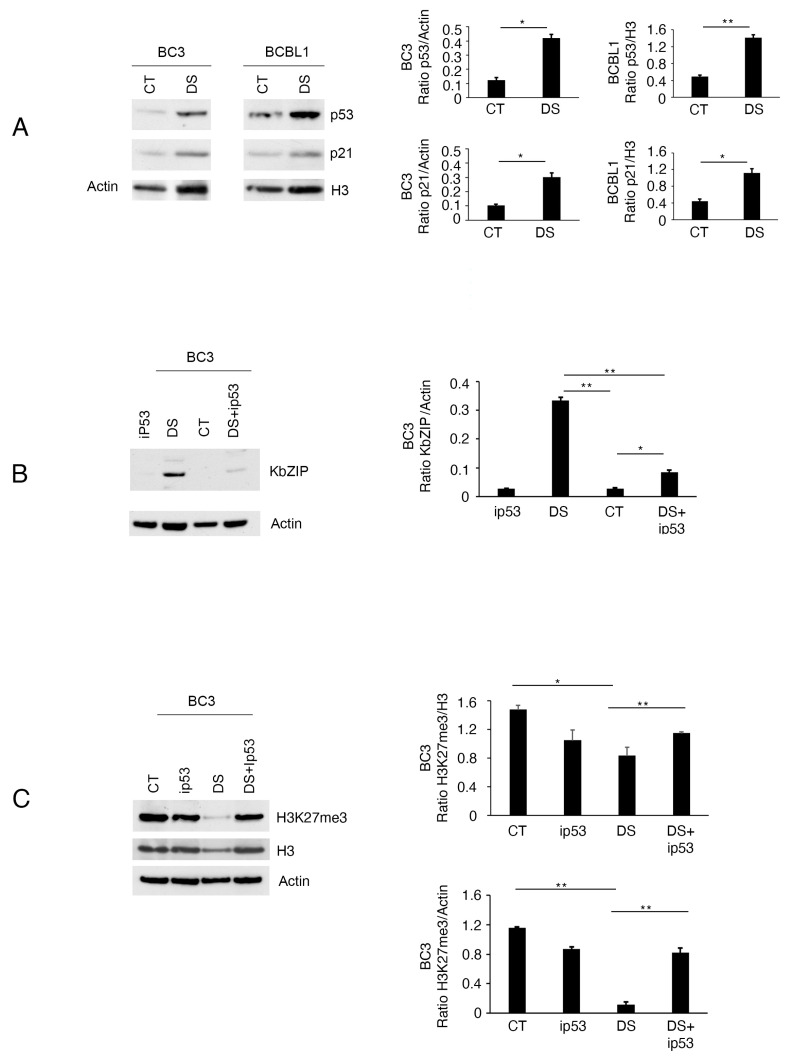
EZH2 inhibition activates wtp53, which contributes to lytic cycle activation and inhibits EZH2 activity. (**A**) p53 and p21 expression levels as evaluated by Western blot in BC3 and BCBL-1 cells treated by DS; (**B**) expression of KbZIP in PEL cells pretreated or not with Pifithrin-α p53 inhibitor (ip53) before exposure to DS; (**C**) H3K27me3 expression in BC3 cells treated by DS or Pifithrin/DS (ip53+DS) combination. Bar-graphs represent the mean plus SD of proteins of interest on house-keeping proteins calculated based on three different experiments. The difference was considered statistically significant when the *p*-value was: * < 0.05; ** < 0.01.

Several studies, performed in our laboratory and others have highlighted that treatments that lead to wtp53 activation promote KSHV replication in PEL cells [15,16,18]. It has also been reported that the depletion of EZH2 may be accompanied by an increase in wtp53 stability [12]. Therefore, here we investigated if DS could activate wtp53 and if its activation could contribute to lytic cycle induction. As shown in Figure 2A, the wtp53 expression level increased, and its target p21 was activated in BC3 and BCBL-1 cells undergoing DS treatment for 24 h, the time at which KbZIP and p64 were expressed (Figure 1B,C). Despite the fact that BCBL-1 carries a monoallelic mutation of p53, we obtained similar results in response to DS treatment, which might suggest that also p73, a transcriptionally active isoform that overlaps with p53 in regulating the expression of p53-dependent genes, could play a role. To find out if the activation of the wtp53–p21 axis could play a role in KSHV lytic cycle reactivation by DS, we pharmacologically inhibited wtp53 by pretreating cells with Pifithrin-α, an inhibitor of p53-dependent transactivation. As shown in Figure 2B, this treatment reduced KbZIP expression in cells treated by DS, suggesting that wtp53 activation contributed to triggering the lytic cycle, according to the above reported studies. As wtp53 has been reported to suppress EZH2 gene expression [13], we then investigated whether wtp53 inhibition could counteract H3K27me3 demethylation mediated by DS. As shown in Figure 2C, the H3K27me3 expression level was restored in the presence of Pifithrin-α in BC3 PEL cells treated by this EZH2 inhibitor. These results suggest that a regulatory circuit between wtp53 activation and H3K27me3 demethylation sustained the KSHV lytic cycle activation in PEL cells treated by DS. This is in agreement with the above reported studies showing that wtp53 inhibited EZH2 [13] and that EZH2 inhibition was able to induce a cytotoxic effect against pancreatic cancer cells only if they carried wtp53, by inducing its activation [12].

### 3.3. DS Activates the First Steps of Autophagy While Inhibiting the Final Steps to Sustain the KSHV Lytic Cycle 

**Figure 3:**
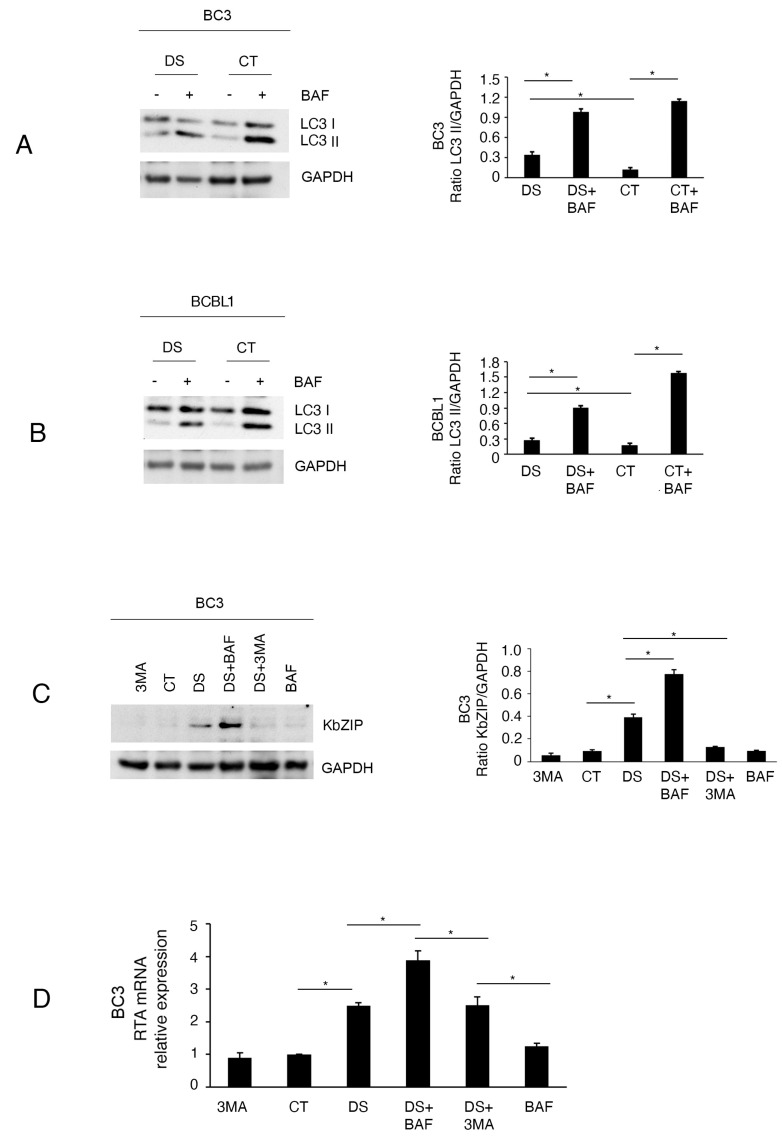
DS triggers autophagy and inhibits its final phases, which promotes the KSHV lytic cycle. (**A**,**B**) Microtubule associated protein 1A/1B light chain 3 (LC3)I/II accumulation in BC3 and BCBL-1 cells treated by DS in the presence or absence of Bafilomycin A (BAF), as evaluated by Western blotting; (**C**) KbZIP expression in BC3 PEL cells treated by DS in the presence or absence of 3-methyladenine (3-MA) or Bafilomycin (BAF). Bar-graphs represent the mean plus SD of proteins of interest on house-keeping proteins calculated based on three different experiments. (**D**) Expression of RTA, as evaluated by RT-qPCR in BC3 cells treated or not with DS in the presence or absence of 3-MA or Bafilomycin (BAF). The difference was considered statistically significant when the *p*-value was: * < 0.05.

Activation of the first autophagic steps has been reported to aid KSHV replication [21], even if the autophagic flux is blocked at the final steps to avoid viral degradation into the lysosomes. Here, we found that DS activated the early phases and reduced the late phases of autophagy, based on the finding that the lipid form of microtubule associated protein 1A/1B light chain 3 (LC3) II slightly increased in BC3 and BCBL-1 PEL cells treated by DS but did not further accumulate in DS-treated cells in the presence of bafilomicin (BAF) compared to those treated with BAF only (Figure 3A,B). BAF is an inhibitor of vacuolar H^+^-ATPase, which alters lysosomal pH and, therefore, blocks autophagy at the final steps, preventing LC3II lysosomal degradation and allowing evaluation of LC3II formation. In agreement with our previous studies [20], here we found that treatment by 3-methyladenine (3-MA), which blocks the first autophagic steps, reduced the expression of KbZIP in BC3 PEL cells treated by DS, while BAF supplementation, which further blocks the late steps of autophagy, increased it (Figure 3C). We then evaluated RTA expression by RT-qPCR in BC3 cells undergoing DS treatment in the presence or absence of 3-MA or BAF and found that the first treatment slightly influenced it, while BAF increased its expression (Figure 3D). Overall, these results suggest that DS activated the initial phases of autophagy while reducing the later stages, an effect that contributed to the activation of KSHV lytic cycle.

### 3.4. EZH2 Inhibition Potentiates Lytic Cycle Activation and the Cytotoxicity Mediated by SAHA

**Figure 4:**
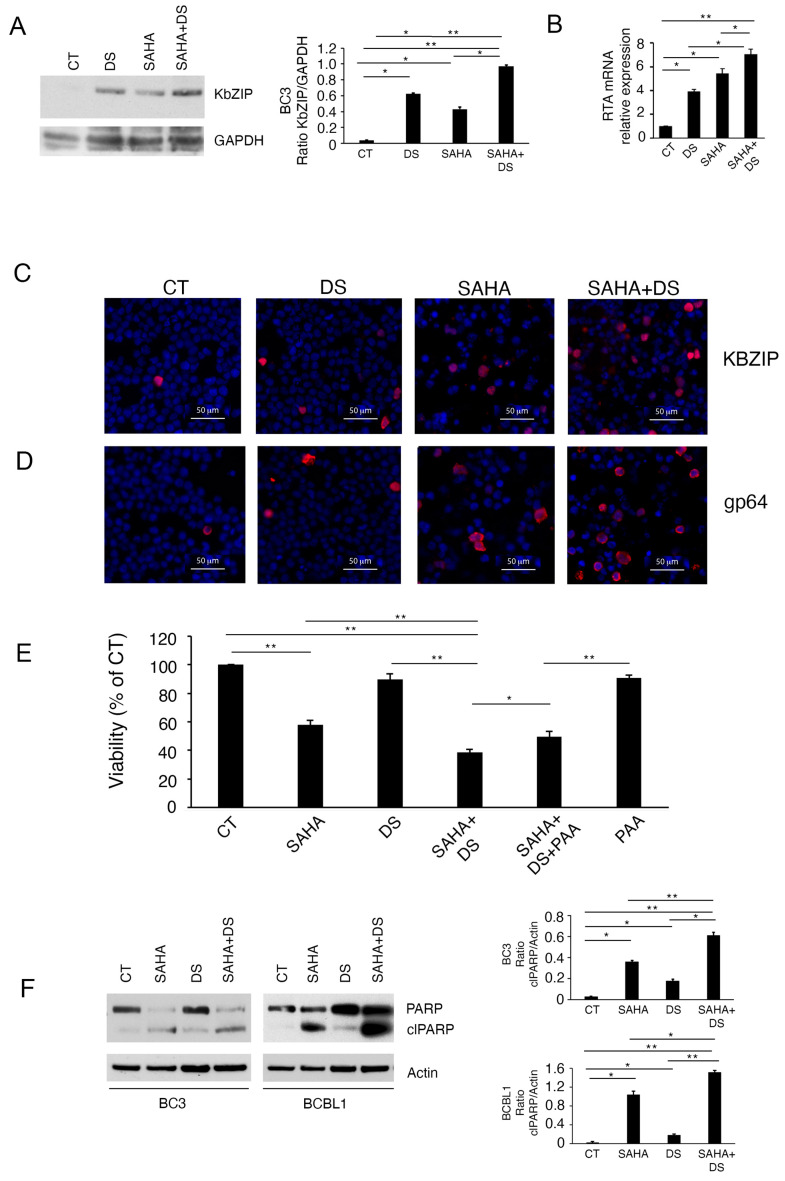
DS increases the activation of the viral lytic cycle and the cytotoxic effect induced by suberoylanilide hydroxamic acid (SAHA). BC3 PEL cells, treated by DS, SAHA or combination of both were investigated (**A**) for the expression of KbZIP by Western blot analysis; (**B**) for the expression of RTA by RT-qPCR, (**C**) for the expression of KbZIP, and (**D**) gp64 by IFA, following treatment by DS in the presence or absence of SAHA; bar = 50 μM. (**E**) Cytotoxic effect of DS, SAHA or combination of both as evaluated by trypan-blue assay in BC3 cells after 24 h of treatment, in the absence or presence of phosphonoacetic acid PAA; (**F**) Poly adenosine diphosphate-ribose polymerase (PARP) cleavage in BC3 and BCBL-1 cells treated by DS, SAHA or DS/SAHA combination investigated by Western blotting. Bar-graphs represent the mean plus SD of proteins of interest on house-keeping proteins calculated based on three different experiments. The difference was considered statistically significant when the *p*-value was: * < 0.05; ** < 0.01.

Histone deacetylases (HDAC) inhibitors are epigenetic drugs known to trigger KSHV replication [5], given that deacetylation represents one of the mechanisms through which viruses silence lytic gene expression [23]. Here we found that the expression of KbZIP was induced by the HDAC inhibitor Suberoylanilide hydroxamic acid (SAHA) and that this effect was potentiated by the concomitant EZH2 inhibition, as evaluated by Western blot (Figure 4A). These results were confirmed by evaluating RTA expression by RT-qPCR (Figure 4B) and by evaluating KbZIP expression by IFA (Figure 4C). We also found that the expression of the p64 late lytic antigen increased in PEL cells following the combination SAHA/DS treatment (Figure 4D). Therefore, this study shows for the first time that the concomitant inhibition of two repressive epigenetic changes induced by KSHV, namely H3K27me3 and histone deacetylation, could synergize in activating the KSHV lytic cycle in PEL cells. Interestingly, DS also potentiated the cytotoxic effect of SAHA in both BC3 and BCBL-1 PEL cell lines (Figure 4E), suggesting that this combination could be used also to efficiently impair PEL cell survival. To evaluate the contribution of lytic cycle activation to cell death induced by SAHA/DS, we pretreated PEL cells with Phosphonoacetic acid (PAA) and found that it slightly influenced the cytotoxic effect of the treatment (Figure 4E). Searching for the type of cell death, we then found that poly adenosine diphosphate-ribose polymerase (PARP) cleavage more strongly increased in BC3 and BCBL-1 cells treated by DS in combination with SAHA (Figure 4F), suggesting the induction of an apoptotic cell death.

## 4. Discussion

**Figure 5:**
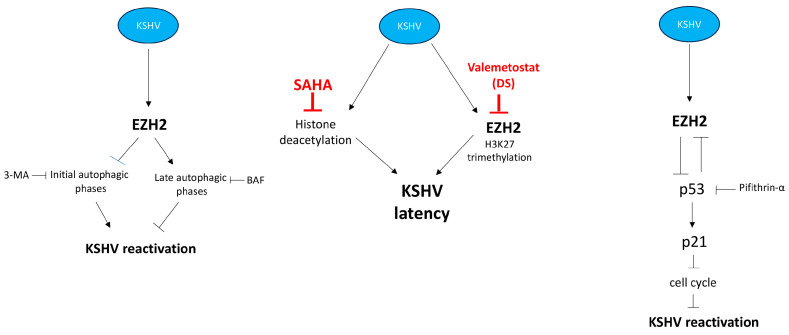
Scheme recapitulating the involvement of EZH2 in KSHV lytic cycle.

In this study, we show that Enhancer of Zeste Homolog 2 (EZH2) inhibition by valemetostat DS3201 (DS) induced the lytic reactivation of Kaposi sarcoma associated herpesvirus (KSHV) from latency in BC3 and BCBL-1 primary effusion lymphoma (PEL) cells and unveil new molecular mechanisms involved in this effect. Indeed, we found that DS activated wtp53 and promoted the initial phases of autophagy, both effects contributing to KSHV lytic antigen expression (Figure 5). Accordingly, EZH2 methyltransferase has been previously shown to regulate the autophagic process [19]. Epigenetic changes are among the most important mechanisms through which viruses, particularly those persisting in the infected host, restrict viral protein expression and maintain latency, which together with other strategies, may help them to avoid immune recognition [24]. Through epigenetic changes, viruses also dysregulate the expression of cellular genes, once infection is established [25]. For example, EZH2 and c-Myc upregulation by human Cytomegalovirus (HCMV) contributes to viral-driven oncogenesis [26,27]. In the specific case of KSHV, it has been reported that the latent protein viral FLICE-inhibitory protein (v-FLIP) upregulated EZH2 in human endothelial cells, which contributed to angiogenesis [28]. 

DNA methylation and histone modifications, including acetylation and methylation, are key events in the complex regulation of gene expression, further complicated by the cross-talk between epigenetic modifications [8]. Interestingly, in this study, we showed that EZH2 inhibition synergized with the histone deacetylases (HDAC) inhibitor suberoylanilide hydroxamic acid (SAHA) in activating the lytic cycle of KSHV in PEL cells. The activation of gammaherpesvirus replication by HDACi has been demonstrated in previous studies, for example following treatment with tricostastin (TSA) or butyrate [23], given that histone deacetylation is known to silence gene expression. As H3K27me3 mark, the target of EZH2 methyltransferase activity, is also associated with KSHV gene repression [11], the combination of SAHA and DS led to a stronger activation of viral lytic cycle in PEL cells. The majority of these cells indeed carry the virus in a latent state, which facilitates viral persistence into the infected host and promotes oncogenesis. Several pro-survival and oncogenic pathways, such as, for example, STAT3 and PI3K/AKT/mTOR, can be activated by KSHV proteins, making this lymphoma particularly aggressive and difficult to treat [29]. However, if the activation of a complete lytic cycle in a small number of cells ensures the survival of the virus and contributes to carcinogenesis, the activation of this process in a large number of cells may lead to lymphoma cell death, given that KSHV replication is accompanied by cell lysis, as for other herpesviruses. Therefore, unbalancing latency/replication towards replication may represent a possible strategy to impair the survival of PEL cells. Interestingly, in this study, both KSHV lytic cycle activation and apoptosis increased in PEL cells following treatment with DS in combination with SAHA. In previous studies, we demonstrated that the first autophagic steps contributed to Epstein-Barr virus (EBV) [20] and KSHV replication [21], and that the autophagic flux was blocked at the final phases to avoid the lysosomal degradation of viral particles entrapped into autophagosomes. Here, based on microtubule associated protein 1A/1B light chain 3 (LC3)II accumulation in PEL cells treated with DS in the presence or absence of BAF, we observed that the inhibition of EZH2 promoted the first autophagic phases and that the autophagic flux was reduced at the final steps. Moreover, bafilomycin supplementation, which further blocked the lysosomal activity in cells treated by DS, increased the expression of KbZIP compared to DS alone, while 3-methyladenine (3-MA,) which inhibits autophagosome formation, counteracted such an effect. In conclusion, this study unveils new molecular mechanisms involved in the activation of the KSHV lytic cycle by EZH2 inhibition in PEL cells, namely, the activation of the wtp53-p21 axis and autophagy dysregulation. The correlation between EZH2 inhibition, p53 activation, autophagy dysregulation and the KSHV lytic cycle activation has not been evaluated in previous studies concerning host chromatin-modifying proteins and latent to lytic transition [30]. Therefore, the findings of the present study may help to shed more light on the mechanisms through which epigenetic drugs can shift the balance from latency to replication and impair the survival of lymphoma cells that harbor this oncovirus in a latent state. 

## Data Availability

The data supporting the conclusions of this article will be made available by the authors on request.
